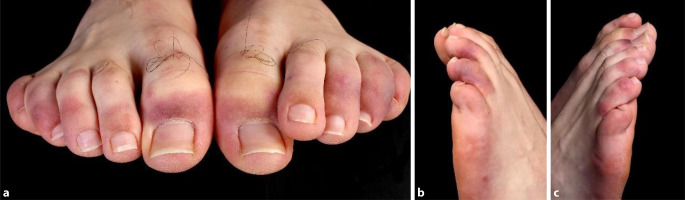# COVID-19 und Hautmanifestationen: Übersicht der aktuellen Literatur im Rückblick auf die bisherige Pandemie

**DOI:** 10.1007/s00105-022-04961-2

**Published:** 2022-03-07

**Authors:** Kristin Lange, Maja Matthies, Parnian Firouzi-Memarpuri, Bernhard Homey

**Affiliations:** Klinik für Dermatologie, Universitätsklinikum Düsseldorf, Moorenstr. 5, 40225 Düsseldorf, Deutschland

**Keywords:** Effloreszenz, Hautausschlag, Haut, Coronavirus, Kutan, Efflorescence, Rash, Skin, Corona virus, Cutaneous

## Abstract

**Hintergrund:**

Die durch das Severe Acute Respiratory Syndrome Corona Virus 2 (SARS-CoV-2) ausgelöste anhaltende globale Pandemie manifestiert sich neben bekannten Organsystemen auch an der Haut. In der Literatur wurden verschiedene klinische Muster von Hauterscheinungen mit COVID-19 in Verbindung gebracht. Die Kenntnis der kutanen Manifestationen kann bei der Früherkennung, Risikostratifizierung von Patienten, Diagnose und den therapeutischen Strategien hilfreich sein. In dem vorliegenden Artikel wird der aktuelle Kenntnisstand zu dermatologischen Befunden im Zusammenhang mit COVID-19 unter Berücksichtigung der klinischen Präsentation, aktuellen pathophysiologischen Konzepten und Management zusammengefasst. Zukünftig sollen auch Erkenntnisse aus einem aktuell geführten Register der Universität Harvard gezogen werden können. Derzeit werden hier Hautveränderungen in Assoziation mit COVID-19 gesammelt (https://ilds.org/covid-19/international-dermatology-covid19-registry/).

**Ziel der Arbeit:**

Ziel dieses Übersichtsartikels ist es, die aktuell verfügbare Literatur mit Hinweisen auf Hautveränderungen im Zusammenhang mit COVID-19 zu analysieren, zu strukturieren und die wichtigsten Aspekte zusammenzufassen.

**Material und Methoden:**

Es wurde eine systematische Literaturrecherche in der medizin-wissenschaftlichen Datenbank PubMed und Medline für englischsprachige Originalartikel, Fallserien und -berichte sowie Übersichtsarbeiten unter Verwendung der Suchbegriffe „Covid“, „COVID-19“ oder „SARS-CoV-2“ in Kombination mit „*skin*“ oder „dermatol“ durchgeführt, welche bis Februar 2021 (Einträge bis zum 28.02.2021) veröffentlicht wurden. Untersucht wurde der Zusammenhang zwischen bestätigten oder vermuteten SARS-CoV-2-Infektionen in Assoziation mit Hautmanifestationen. Eingeschlossen wurden vorwiegend Arbeiten, welche ein möglichst großes Patientenkollektiv, das Erwachsenenalter und einen positiven Corona-Nachweis beinhalteten. Ziel der Arbeit ist es, einen Überblick der am häufigsten beobachteten Hautmanifestationen bei Infektionen mit SARS-CoV‑2 zu geben.

**Ergebnisse und Diskussion:**

Die Zuordnung der Hautmanifestationen im Rahmen einer SARS-CoV-2-Infektion nach klinisch dermatologischen Mustern kann dazu beitragen, Patienten mit erhöhtem Risiko frühzeitig zu identifizieren und adäquat zu behandeln, um einem möglicherweise schwereren Krankheitsverlauf wie er beispielsweise bei der Livedo auftritt, entgegenzuwirken. Die erworbenen Kenntnisse der pathophysiologischen Mechanismen können zu einem verbesserten Management der Erkrankung und Hilfestellung möglicher Gegenmaßnahmen in der Bewältigung der Erkrankung beitragen.

## Abstract (en)

**Background:**

The persistent global pandemic caused by severe acute respiratory syndrome coronavirus 2 (SARS-CoV-2) can manifest on the skin in addition to the already known organ systems. Various clinical patterns of skin manifestations associated with coronavirus disease 2019 (COVID-19) have been described. In view of the associated morbidity and mortality, knowledge of cutaneous manifestations in the setting of COVID-19 may be helpful in early detection, risk stratification, diagnosis and treatment. Thus, we provide a summary of the various dermatologic findings associated with COVID-19, including clinical presentation, current pathophysiological concepts, and management, to support early diagnosis and treatment.

**Goal:**

The current literature regarding skin lesions associated with COVID-19 and the most important aspects are analyzed.

**Materials and methods:**

A literature search was performed in PubMed and Medline databases until February 28, 2021.

**Results and conclusion:**

The classification of skin manifestations in the context of SARS-CoV‑2 infection according to clinical dermatological patterns can help to identify patients with increased risk at an early stage and to treat them adequately to counteract a possibly more severe course of the disease as it occurs, e.g., in livedo. Thus, knowledge of the pathophysiological mechanisms can improve management of the disease and support possible countermeasures in coping with the disease.

Die ersten Berichte von Patienten, welche sich mit dem neuartigen Coronavirus infiziert haben, stammen aus der Region Wuhan, China, von Dezember 2019 [31]. Im März 2020 wurde SARS-CoV‑2 offiziell durch die Weltgesundheitsorganisation zur globalen Pandemie deklariert [1, 11]. Ein Jahr nach Ausbruch der weltweiten Pandemie sind die Erkenntnisse zur Infektion mit SARS-CoV‑2 und damit verbundenen gesundheitlichen Folgen, welche sich aus Literatur und gesammelter klinischer Erfahrung ergeben, ausgeweitet. Neben den unterschiedlichsten Organmanifestationen und führenden Symptomen wurden in einer Reihe von Fallberichten und Metaanalysen unterschiedliche Hautmanifestationen beschrieben. Die Inzidenz kutaner Manifestationen assoziiert mit COVID-19 war in den bisher berichteten Studien sehr heterogen und reichte von nur 0,2 % bis zu 20,4 % [19, 33]. Wie bereits anhand der uneinheitlichen Prävalenzen zu erahnen, lässt sich nur schwierig beurteilen, ob sich bei den beschriebenen Hautveränderungen ein kausaler Zusammenhang mit COVID-19 herstellen lässt oder es sich um unspezifische Assoziationen im Sinne von virusbedingten Exanthemen handelt. Erschwerend für die Beurteilung kommt neben der großen Varianz der Inzidenz kutaner Manifestationen hinzu, dass häufig kein direkter, positiver Nachweis einer Infektion mit SARS-CoV‑2 erfolgen konnte. Insofern sollten die Beschreibungen von Hautveränderungen in Zusammenhang mit COVID-19 in der Literatur und im klinischen Alltag kritisch hinterfragt werden. Konkrete Rückschlüsse sollten trotz der zahlreichen Berichte nicht ohne Weiteres abgeleitet werden, da die aktuelle Datenlage für eine evidenzbasierte Aussage derzeit nicht ausreichend erscheint, eine systematische Analyse liefert aber erste wichtige Hinweise. Basierend auf der aktuellen Literatur, findet sich ein Konsens unter mehreren Autoren, welche die Hautmanifestationen zur weiteren Einordnung verschiedenen klinisch-dermatologischen Mustern zugeordnet haben und somit bedingt Hinweise zu Diagnostik, Verlauf und Schweregrad der Erkrankung liefern.

## Klinisch-dermatologische Muster der COVID-19-Infektion

**Figure 1:**
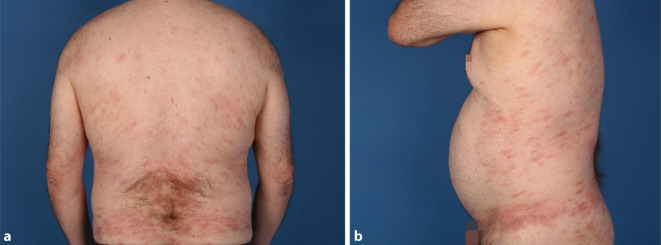

Die italienische Arbeitsgruppe um Sebastiano Recalcati aus Lecco berichtete erstmals von Hautmanifestationen bei Patienten, welche sich mit SARS-CoV‑2 infiziert hatten. Zirka 20 % der eingeschlossenen Patienten zeigten eine kutane Beteiligung im Rahmen einer SARS-CoV-2-Infektion [33]. Dabei traten erste Hautveränderungen häufig nach den Symptomen der Infektion mit unterschiedlicher Latenz auf. Seltener wurden Hautveränderungen beobachtet, welche sich simultan oder in der Prodromalphase der Infektion zeigten. Verschiedene dermatologische Muster wurden bisher diskutiert. Recalcati et al., Marzano et al. und Rahimi et al. identifizierten die folgenden 6 dermatologischen Muster: 1) makulopapulöses Exanthem (Abb. 1), 2) urtikarielles Exanthem, 3) Chilblain-ähnliches Akralmuster (Abb. 2), 4) vesikulöses Exanthem, 5) Livedo-ähnliche Läsionen (Livedo reticularis, Livedo racemosa) und 6) purpurische „vaskulitische“ Läsionen [27, 32, 33].

## Makulopapulöse Exantheme

Den größten Anteil an COVID-19 assoziierten Hautveränderungen stellen exanthematische Hautveränderungen dar. Im Kollektiv von Galván et al. fand sich bei 47 % der 375 eingeschlossenen Patienten ein makulopapulöses Exanthem, welches in über 50 % mit Juckreiz assoziiert war. Die Exantheme variierten in Form und Auftreten stark. Laut einer Übersichtsarbeit von Shams et al. mit 354 Patientenfällen mit exanthematischen Hautveränderungen traten diese mit einem Durchschnittsalter von 53 Jahren auf und dauerten 8 Tage an [35]. Als Hauptlokalisation wurde der Körperstamm beschrieben, gefolgt von den Extremitäten [16, 35]. Die Hautveränderungen traten überwiegend nach den ersten COVID-19-typischen respiratorischen Symptomen auf. Als therapeutische Maßnahmen erfolgten je nach Schweregrad die Gabe von Antihistaminika und topischen Glukokortikosteroiden. Bei ausgeprägten Verläufen wurden auch systemische Glukokortikosteroide verabreicht. Zu beachten ist bei der Bewertung, dass viele Patienten auch systemische Therapien mit beispielsweise Makroliden erhielten. Eine abzuwägende Differenzialdiagnose ist daher das Arzneimittelexanthem [35]. Histopathologisch zeigten sich vorwiegend unspezifische Entzündungsreaktionen mit superfizieller perivaskulärer Dermatitis mit lymphozytärem Infiltrat sowie erweiterten Gefäßen in der papillären und mittleren Dermis mit Neutrophilen, Eosinophilen und nukleärem Detritus. Pathophysiologisch sind 2 Hypothesen zu beachten: die Entstehung eines Exanthems durch die direkte Infektion der Haut gegenüber einem sekundären Effekt, ähnlich zur Entstehung eines postinfektiösen Exanthems durch virusinduzierte Immunaktivierung [4]. Da auch andere Virusinfektionen mit Exanthemen einhergehen, ist eine ähnliche Genese denkbar. Die Hypothese, dass Exantheme durch direkte Wirkung des Virus entstehen könnten, wird gestützt durch Untersuchungsergebnisse aus der Arbeitsgruppe um Shams: Hier wurden in T‑Zellen Partikel des SARS-CoV-2-Virus sowie RNA-Teile gefunden [35]. Es ist also von einer direkten Infektion der T‑Zellen durch das Virus auszugehen, resultierend in einer vermehrten Freisetzung von Zytokinen wie Interleukin‑6. Eine lokale übermäßige Freisetzung von Zytokinen in die Haut kann zu einer Aktivierung von dermalen dendritischen Zellen, Lymphozyten, Makrophagen, Mastzellen und Neutrophilen führen und folglich ein makulopapulöses Exanthem auslösen.

## Urtikaria/Angioödeme

In der Literatur wird die Urtikaria als eine häufige Hautmanifestation im Rahmen einer Infektion mit SARS-CoV‑2 beschrieben [2]. Multiple Einflüsse wie bakterielle oder virale Infektionen, insbesondere des oberen Respirationstrakts, Medikamenteneinnahme sowie die Kombination dieser Faktoren können ursächlich für die Entwicklung einer akuten Urtikaria sein, sodass eine SARS-CoV-2-Infektion keine Ausnahme darstellt [3]. Bei den meisten Patienten in der vorliegenden Literatur trat die Urtikaria gleichzeitig oder bis zu 48 h vor krankheitstypischen Symptomen von COVID-19 auf, welches die Bedeutung der Früherkennung von Urtikaria bei der Diagnosestellung und Krankheitsausbreitung von COVID-19 unterstreicht. Galván Casas et al. fanden bei 73 von 375 Patienten urtikarielle Hautveränderungen [16]. Die italienische Arbeitsgruppe um Recalati et al. fand bei 3 von 18 Patienten das Bild einer Urtikaria im Rahmen einer SARS-CoV-2-Infektion [33]. Die Prävalenz der Urtikaria unter anderen beschriebenen kutanen, COVID-19-assoziierten Manifestationen variierte in kleineren Fallserien zwischen 7 % und 40 %. Die große Diskrepanz der beschriebenen Prävalenzen spiegelt eine mögliche uneinheitliche Beschreibung der Hautveränderungen durch verschiedene Autoren wider. Nicht immer ist eine Differenzierung zwischen Urtikaria und urtikariellen Exanthemen, die auch das urtikarielle Arzneimittelexanthem einschließen, deutlich für den Leser ersichtlich. Insbesondere bei Patienten mittleren Alters traten urtikarielle Läsionen gehäuft auf [34]. Laut aktueller Datenlage rezidivierten urtikarielle Hautläsionen durchschnittlich über einen Zeitraum von 1 Woche und verschwanden häufig mit Abklingen anderer Symptome der Infektion [21]. Die Hautveränderungen fanden sich hauptsächlich am Köperstamm lokalisiert, seltener im Gesichtsbereich oder an den Akren. Sehr häufig waren sie mit ausgeprägtem Juckreiz (92 %) verbunden [15]. Auf eine Therapie mit Antihistaminika, topischen und systemischen Glukokortikosteroiden zeigte sich ein sehr gutes Ansprechen [40]. Differenzialdiagnostisch sollten andere mögliche Infektionen und Arzneimittelreaktionen bedacht werden, insbesondere müssen kutane Nebenwirkungen eingesetzter potenzieller Anti-COVID-19-Medikamente wie Chloroquin, Hydroxychloroquin und Lopinavir, Ritonavir und Nitazoxanid berücksichtigt werden. Ein urtikarielles Arzneimittelexanthem konnte in vielen Fällen nicht sicher ausgeschlossen werden [37]. Histopathologisch wird das typische Bild einer Urtikaria mit perivaskulärem lymphozytärem Infiltrat mit einzelnen Eosinophilen sowie dermalem Ödem und Spongiose beschrieben. Bei Läsionen mit Urtikaria-Vaskulitis-ähnlichem Aspekt zeigten sich eine Blutextravasion und ein perivaskuläres neutrophilenreiches entzündliches Infiltrat mit Fragmentierung der Zellkerne [23].

Obwohl bei Patienten mit SARS-CoV-2-Infektion urtikarielle Läsionen häufiger beobachtet wurden, war ein assoziiertes Angioödem nur selten beschrieben. Hassan et al. berichteten über eine 46-jährigen Frau, welche sich mit einer akuten Urtikaria und Angioödem vorstellte und erst am zweiten Krankheitstag respiratorische Symptome wie trockenen Husten und Fieber entwickelte [20]. Cohen et al. beschrieben einen Fall eines 61-jährigen Mannes mit einer SARS-CoV-2-Infektion, welcher einen Tag vor Symptomen (Fieber, Schüttelfrost, Husten) im Rahmen von COVID-19 eine Lippen- und Gesichtsschwellung aufwies. Andere Hautläsionen oder Juckreiz waren nicht vorhanden. Es kam zu einer Besserung der Symptomatik innerhalb von 2 Tagen nach Gabe von Methylprednisolon und Diphenhydramin [9]. Pathophysiologisch könnte eine plausible Erklärung für die Entwicklung eines Angioödems in der etablierten Korrelation zwischen SARS-CoV‑2 und dem Angiotensin-Converting-Enzym 2, einem *Entry*-Rezeptor für das Virus, das in den Epithelzellen der Lunge zu finden ist, liegen. Bereits bekannt ist, dass das Angiotensin-Converting-Enzym 2 eine entscheidende Rolle bei der Hemmung von Des-Arg9-Bradykinin, einem potenten Liganden des Bradykinin-Rezeptors 1 spielt. Daher führt die Hemmung des Angiotensin-Converting-Enzyms 2 zu einer übermäßigen Aktivierung des Bradykinin-Signalwegs und erhöht anschließend die Gefäßpermeabilität. Eine exzessive Bradykinin-Produktion gilt als Schlüsselmechanismus in der Pathogenese des Bradykinin-vermittelten Angioödems. Dieses Modell ähnelt dem vermuteten Mechanismus, durch den das Virus ein akutes Lungenödem und Atemnotsyndrom verursacht [13, 22, 30, 38, 43].

## Vesikulöse Exantheme

Vesikulöse Exantheme als spezifische COVID-19-assoziierte Hautmanifestationen wurden erstmalig durch Marzano et al. und Galván et al. beschrieben [16, 28]. In der Literatur wurde das Auftreten mit Prävalenzen zwischen ~4 % und 15 % angegeben [28]. Es wird angenommen, dass vesikulöse Läsionen mit einem mäßigen Schweregrad der Erkrankung verbunden sind [16, 27]. In der großen prospektiven spanischen Studie fand sich ein vesikulöses Exanthem bei 9 % der 375 untersuchten Patienten [16]. Vesikulöse Läsionen fanden sich typischerweise bei Patienten mittleren Alters. Dabei traten die Läsionen im Durchschnitt 3 Tage nach anderen COVID-19 typischen Symptomen auf mit einer mittleren Verweildauer von 8,4 Tagen und heilten ohne Ausbildung von Narben komplikationslos ab. Vesikulöse Eruptionen wurden auch während der asymptomatischen Phase beobachtet. Als typische Lokalisation wurde am häufigsten der Körperstamm beschrieben, gefolgt von den Extremitäten [5, 36]. Das klinische Bild der vesikulösen Läsionen in COVID-19-Studien variierte stark von diffusen polymorphen bis zu lokalisierten monomorphen Verteilungsmustern, teils mit hämorrhagischem Inhalt. Eine prospektive Studie von Fernandez-Nieto et al. konnte die vesikulösen Hautveränderungen weiter charakterisieren: Von 22 Patienten mit vesikulösen Läsionen zeigten 75 % ein diffuses Muster polymorpher Läsionen und 25 % ein lokalisiertes Muster monomorpher Läsionen am Rumpf [14]. Als mögliche Begleitsymptome wurden häufig Juckreiz, seltener Schmerzen oder Brennen angegeben. Zwei von 130 Patienten hatten während ihres stationären Aufenthalts isolierte herpetiforme Läsionen am Körperstamm [36].

Histopathologische Untersuchungen wurden unter anderem in 2 Studien durchgeführt, und beide beschrieben ihre Ergebnisse als konsistent mit bekannten Virusinfektionen. Dabei zeigte sich in den Hautbiopsien das Vorhandensein von intraepidermalen Vesikeln, die mit milder Akantholyse und ballonierten Keratinozyten assoziiert waren [14]. Marzano et al. fanden in den histologischen Untersuchungen bei 7 Patienten mit einer Hautbiopsie das Bild einer „Korbwellenhyperkeratose“, eine leicht atrophe Epidermis und vakuolige Degeneration der Basalzellschicht mit mehrkernigen, hyperchromatischen Keratinozyten und dyskeratotischen Zellen [28]. Mögliche Theorien bezüglich pathophysiologischen Mechanismen schließen laut Criado et al. eine Überaktivierung des Immunsystems, resultierend in einem potenziellen Zytokinsturm mit Beteiligung der Haut, ein. Dieselbe Arbeitsgruppe spekulierte, dass eine direkte zytopathische Wirkung von SARS-CoV‑2 auf Endothelhautgefäße vesikulöse Läsionen hervorrufen könnte [10]. Im Gegensatz zu makulopapulösen und urtikariellen Hautveränderungen werden vesikulöse Läsionen bei COVID-19 weniger häufig als ätiologisch mit antiviralen Arzneimitteln oder anderen Therapien verbunden angesehen. Jedoch sollte insbesondere im Hinblick auf die Histologie eine schwere Arzneimittelreaktion berücksichtigt werden, welche sich klinisch auch als vesikulöse Hautveränderung präsentieren kann wie beispielsweise im Rahmen einer toxisch epidermalen Nekrolyse (TEN).

## Chilblain-artige Hautveränderungen

In der Literatur wurden wiederholt „Frostbeulen“-ähnliche, akrale Hautveränderungen im Zusammenhang mit einer möglichen SARS-CoV-2-Infektion beschrieben. Es scheint sich um eine spezifische Hautveränderung zu handeln, welche laut Beobachtungen der spanischen Arbeitsgruppe um Marzano fast ein Fünftel (19 %) der beobachteten Hautveränderungen einnimmt [27]. Überwiegend betroffen waren die Füße mit bis zu 1 cm messenden livid-erythematösen Papeln und Nodi. Die Hautveränderungen traten insbesondere im späteren Krankheitsverlauf auf, meist nach Krankheitshöhepunkt [16] und waren oft asymptomatisch oder nur wenig symptomatisch, mit leichtem Juckreiz oder brennenden Schmerzen einhergehend [6]. Es fanden sich selten Hinweise auf „Frostbeulen“ in der Krankheitsvorgeschichte, sodass die Hautveränderungen überwiegend erstmanifestierend waren. Freeman et al. dokumentierten bei 18 % der Patienten Chilblain-artige Hautveränderungen, welche durchschnittlich über 14 Tage persistierten [15]. Häufig war ein junges Patientenkollektiv (19,9 Jahre) mit leichten COVID-19 spezifischen Symptomen betroffen. In der Arbeitsgruppe um Bouaziz wurden die Hautveränderungen zudem bei 40 Patienten mit unklarem oder negativem COVID-19-Status beobachtet [12]. Aufgrund der meist asymptomatischen Hautveränderungen und des selbstlimitierenden Verlaufes war eine Therapie häufig nicht notwendig. Einige Autoren berichteten von der erfolgreichen topischen Anwendung von Glukokortikosteroiden oder auch Vasodilatatoren wie beispielsweise Nitroglycerin [29, 41]. Histologisch zeigten sich die Chilblain-artigen Hautveränderungen laut der Übersichtsarbeit von Kaya et al. als diffuses dichtes lymphozytäres Infiltrat der oberflächlichen und tiefen Dermis mit einem perivaskulären Muster und Zeichen endothelialer Aktivierung [23]. Eine erste Hypothese zur Pathogenese der Hautveränderungen schließt eine durch das SARS-CoV-2-Virus ausgelöste Immunkomplexbildung mit folgender Vaskulitis und Thrombosierung von kleinen Gefäßen ein [7]. Die Hautveränderungen sind vor diesem Hintergrund als eine überschießende Immunreaktion auf das Virus anzusehen. Unterstützend hierfür fand die Arbeitsgruppe um Lee et al. in histologischen Proben der Chilblain-artigen Hautveränderungen Hinweise darauf, dass in den Läsionen bei Kontakt zu SARS-CoV‑2 eine schnelle und lokale Abwehr stattfindet: Sie fanden erhöhte Konzentrationen von Proteinen und Kinasen, welche durch Typ-I-Interferon aktiviert werden [24]. Typ-I-Interferon als Teil des angeborenen Immunsystems spielt eine wichtige Rolle in der Abwehr von Viren. Bei hohen Konzentrationen ist also von einer intakten und schnellen Abwehr auszugehen, was die kurzen und milden Krankheitsverläufe der betroffenen Patienten erklären würde.

## Petechien

In mehreren Arbeiten wurde auch das seltene Auftreten von purpuriformen Hautveränderungen beschrieben. Zumeist handelte es sich dabei um Petechien, welche bei Erwachsenen mit schweren Verläufen der SARS-CoV-2-Infektion assoziiert sind. Galván et al. fanden bei 9 % der eingeschlossenen Patienten das Auftreten von Gefäßeruptionen [16]. Die Effloreszenzen traten zu verschiedensten Zeitpunkten der Infektion auf und zeigten sich betont an Körperstamm und Gesäß. In der Arbeit von Zhao et al. wird die Häufigkeit der Petechien mit 1,58 % beschrieben [42]. Histologisch werden Petechien von Calvao et al. am Beispiel von einem 81-jährigen Patienten mit COVID-19-typischen Symptomen, aber negativer PCR beschrieben: Es zeigten sich neben einem milden inflammatorischen Infiltrat der papillaren Dermis, bestehend aus Neutrophilen und Erythrozytenextravasion, eine Vaskulitis der kleinen Gefäße ohne Thrombosierung und ein dermales Ödem [8]. Pathophysiologisch hypothesierten Valtueña et al. das Vorliegen einer obliterativen Mikroangiopathie aus endothelialem und intensivem myointimalem Wachstum mit Komplementaktivierung [39]. In Zusammenschau mit einer erhöhten Gefäßpermeabilität könnte diese für obliterierte Gefäßlumen und resultierende Hämorrhagie verantwortlich sein. Magro et al. berichten von 5 schwer erkrankten Patienten, bei welchen sich eine Thrombosierung der kleinen Lungengefäße fand [25]. Drei dieser Patienten zeigten zudem Petechien oder livedoähnliche Hautzeichnungen. Es konnte sowohl in den kleinen Gefäßen der Lunge als auch in denen der Haut eine Komplementaktivierung sowie das SARS-CoV-2-spezifische *Spike-*Protein nachgewiesen werden. Dies wurde sowohl in betroffener als auch unauffälliger Haut gefunden. Magro et al. folgerten hieraus, dass bei schwerer Erkrankung ein generalisiertes mikrovaskuläres Verletzungssyndrom vermittelt durch intensive Aktivierung von alternativen Komplement- und Lektinwegen ausgelöst wird und in einem prokoagulativen Status resultiert. Eine weitere Hypothese zur Pathogenese wird von Tammaro et al. postuliert. Die Arbeitsgruppe schreibt den Gefäßschaden dem Virus direkt durch seine Bindung an den Rezeptor des Angiotensin-Converting-Enzym 2 zu, welcher in Endothelzellen vertreten ist. Zudem spekulierten sie, dass eine schwere Infektion zu einer disseminierten intravasalen Koagulopathie führen und damit die Hautschäden verursachen könnte. In der Literatur fanden sich keine spezifischen Therapien. Einige Patienten wurden zu unterschiedlichen Zeitpunkten der Erkrankung antikoaguliert [24].

## Livedo/akrale Ischämien

Weitere beobachtete Hautveränderungen stellen livid-erythematöse, netzartige Muster im Sinne einer Livedo reticularis dar. Oft zeigten sich die Hautveränderungen transient [26]. Im großen Kollektiv von Galván et al. fanden sich die Livedo-Zeichnungen und akrale Ischämien bei 6 % der Patienten [16]. Diese Ergebnisse lassen sich ebenfalls in den Arbeiten von Zhao et al. und Gisondi et al. reproduzieren [18, 42]. Die Arbeitsgruppe um Gisondi bemerkte zudem ein spätes Auftreten der Hautveränderungen und eine extremitätenbetonte Lokalisation. Vorwiegend wurden die Hautveränderungen bei älteren Patienten mit schwerer SARS-CoV-2-Infektion beobachtet. In dieser Patientengruppe fand sich mit 10 % die höchste Mortalität, sodass Livedozeichnungen und Akroischämien als ungünstiger prognostischer Faktor gewertet wurden. Auch Gianotti et al. vermuteten aufgrund schwerer Verläufe, dass diffuse Livedo-artige Hautveränderungen mit einem Multiorganversagen assoziiert sein können [17]. Histologisch beschrieben sie Langerhans-Zell-Nester in der Epidermis und tiefer in der Dermis liegende Mikrothromben mit nukleärem und eosinophilem Detritus bei 2 intensivpflichtigen Patienten mit Livedo-artigen exanthematischen Hautveränderungen.

Die Unterscheidung zwischen Livedo reticularis und Livedo racemosa ist in den gesammelten Artikeln nicht nachzuvollziehen und erlaubt daher anhand der beschriebenen Klinik keinen Rückschluss darauf, ob eher ein Gefäßverschluss oder eine generelle Minderperfusion vorliegt. Mehrere Autoren hypothesieren dennoch die zugrunde liegende Pathophysiologie. Einige führten diese Hautveränderungen auf eine systemische okklusive Gefäßkrankheit zurück. Lee et al. diskutierten, dass es sich pathophysiologisch entweder um primäre, virusassoziierte Läsionen oder sekundäre Hautveränderungen infolge von Gefäßverschlüssen und damit assoziierten Komplikationen handelt [24]. Da Veränderungen der Gerinnung während der Infektion häufig beobachtet wurden, können diese als ursächlich für den Gefäßschaden angesehen werden. Alternativ kann auch hier die überschießende Immunantwort verantwortlich für die Entstehung der Livedo-artigen Hautveränderungen sein.

## Fazit

- Beschriebene Hautveränderungen können wichtige Hinweise auf eine Infektion liefern, da beispielsweise Urtikae vereinzelt auch vor anderen typischen Symptomen auftreten können.
- Ferner können sie einen Beitrag zur Prognoseabschätzung leisten. So wird das Auftreten einer Livedo als ungünstiger prognostischer Marker gewertet.
- Aufgrund der facettenreichen Erscheinungen der Effloreszenzen sollte differenzialdiagnostisch bei Patienten mit vaskulitischen und exanthematischen Hautveränderungen eine Infektion mit SARS-CoV‑2 in Betracht gezogen werden.
- Die Differenzialdiagnosen des Arzneimittelexanthems und anderer virusassoziierter Exantheme sollten stets bei der Begutachtung der Hautveränderungen während der Pandemie berücksichtigt werden.
- Es bedarf weiterer Untersuchungen: vollständige PCR- oder serologische Analyse sowie Hautbiopsien, um die Beziehung zwischen kutanen Symptomen und SARS-CoV‑2 noch besser einordnen und deren Relevanz bewerten zu können.
- Denkbare Marker könnten neben den PCR-basierten Nachweisen auch immunhistochemische Marker beispielsweise für das gut charakterisierte Spike-Protein einschließen.
- Das in Harvard erstellte Register zur Dokumentation wird zu einem besseren Verständnis der Pathogenese, der Krankheit sowie der diagnostischen und therapeutischen Möglichkeiten zukünftig beitragen (https://ilds.org/covid-19/international-dermatology-covid19-registry/).

**Figure 2:**